# Incidence and Risk Factors of Perianal Pathology during Pregnancy and Postpartum Period: A Prospective Cohort Study

**DOI:** 10.3390/jcm13082371

**Published:** 2024-04-18

**Authors:** Zivile Sabonyte-Balsaitiene, Tomas Poskus, Eugenijus Jasiunas, Diana Ramasauskaite, Grazina Drasutiene

**Affiliations:** 1 Clinic of Obstetrics and Gynaecology, Institute of Clinical Medicine, Vilnius University Faculty of Medicine, 03101 Vilnius, Lithuania; diana.ramasauskaite@santa.lt (D.R.); grazina.drasutiene@mf.vu.lt (G.D.); 2 Clinic of Gastroenterology, Nephrourology and Surgery, Institute of Clinical Medicine, Vilnius University Faculty of Medicine, 03101 Vilnius, Lithuania; tomas.poskus@santa.lt; 3 Centre for Informatics and Development, Vilnius University Hospital Santaros Klinikos, 08661 Vilnius, Lithuania; eugenijus.jasiunas@santa.lt

**Keywords:** pregnancy, hemorrhoidal disease, perianal diseases, risk factors

## Abstract

Objective: We aimed to identify the incidence and risk factors of perianal pathology during pregnancy and the postpartum period. Methods: A prospective cohort study was conducted in three institutions in Lithuania. A total of 190 patients were examined and interviewed three times (<12, 18–20 weeks of gestation, and during the first 2 months after delivery). They completed a questionnaire including demographic, obstetric, coloproctological, and birth data. Results: A total of 73 (34.59%) women developed hemorrhoidal disease after delivery, and 120 (56.87%) developed perianal pathology. Multivariate analysis identified a neonatal birth weight ≥3380 g (OR 4.22; 95% CI 1.83–9.71, *p* < 0.001) and consumption of eggs (OR 3.10; 95% CI 1.13–8.53, *p* = 0.028) or cereals (OR 2.87; 95% CI 1.32–6.25, *p* = 0.008) several times per week as significant risk factors for hemorrhoidal disease. Neonatal birth weight ≥3380 g (OR 3.95; 95% CI 1.47–10.59, *p* = 0.006), maternal BMI ≥ 21.48 (OR 3.58; 95% CI 1.51–8.47, *p* = 0.004), the duration of the second labor period ≥38 min (OR 2.81; 95% CI 1.09–7.23, *p* = 0.032), and consumption of flour products several times per week (OR 2.77; 95% CI 1.10–6.98, *p* = 0.030) were associated with a higher risk of perianal pathology. Daily consumption of fruits and vegetables (OR 0.35; 95% CI 0.15–0.81, *p* = 0.014) and less frequent consumption of eggs were protective factors (OR 0.18; 95% CI 0.06–0.56, *p* = 0.003). Conclusions: Perianal diseases, especially hemorrhoidal disease, are common during pregnancy and the postpartum period. A neonatal birth weight ≥ 3380 g, a maternal BMI of ≥21.48, duration of the second labor period of ≥38 min, and consumption of flour products and cereals several times a week are risk factors for developing these diseases.

## 1. Introduction

Perianal diseases are common and can require referral to gastroenterologists and proctologists. The incidence of these conditions in the general Western population ranges from 4% to 10% [1]. The most common benign anal conditions include hemorrhoidal disease (HD), anal fissures, perianal abscesses, fecal incontinence, functional rectal pain, anal itching, and perineal prolapse [2,3].

HD is a common complaint in the general population which is more prevalent in the 45–65-year-old population and women of childbearing age [4]. This condition often develops during pregnancy and the postpartum period [5,6]. The incidence varies from 15% to 41% [7]. Clinical reports have shown that HD is most widespread in the last trimester of pregnancy and the first month after delivery, and approximately 25–35% of pregnant women suffer from it [8].

Pregnancy and childbirth are known to be some of the most critical risk factors for HD and perianal pathologies. The actual cause of HD remains unknown, but there are several theories that physiological factors may trigger it in pregnancy. At the end of pregnancy, the mechanical effect of the enlarging uterus on the digestive system is most pronounced [9]. The growing uterus affects intestinal movements, especially of hard stool, making bowel movements difficult and causing constipation [9,10]. In addition, the increase in intra-abdominal pressure due to the enlargement of the uterus leads to venous stasis in the perianal region and decreased blood circulation to the internal anal sphincter [7,11]. Furthermore, the 25–40% increase in circulating blood volume leads to vasodilatation and venous stasis in the pelvis [11]. During pregnancy, certain hormonal factors also favor the development of disease. A myorelaxant effect characterizes the increasing amount of progesterone in the blood serum. It inhibits the movement of calcium ions in smooth muscle cells and slows down the motility of the entire digestive system [12]. Furthermore, a higher concentration of progesterone in blood serum is associated with 30–50% slower peristalsis in the stomach and intestines [13]. The longest duration of intestinal peristalsis is observed in the second and third trimesters of pregnancy [14].

Some risk factors for perianal pathology and HD have already been demonstrated and recognized in several prospective studies. These include constipation, diarrhea, pregnancy, and delivery [11]. Poskus et al. reported that a personal history of perianal disease, straining during delivery for more than 20 min, newborn birth weight >3800 g, and constipation are independent risk factors for HD and anal fissures [5]. Ferdinande et al. found that constipation and a history of anal problems are significant risk factors for developing perianal disease during pregnancy [15]. Other reported risk factors in pregnant women include obesity and overweight before pregnancy, dyschezia, smoking, unhealthy diet, and a family history of oncologic diseases of the digestive system [6,15,16,17,18]. This study aimed to identify the most important risk factors for the development of HD and perianal pathology in pregnant women to assist in the possible prevention of those conditions during pregnancy and after delivery.

## 2. Materials and Methods

### 2.1. Study Design

A prospective cohort study was carried out in 3 hospitals (Vilnius University Hospital Santaros Klinikos, Vilnius City Clinical Hospital, and Vilnius Maternity Hospital) in Lithuania. Participants were enrolled in the study between June 2016 and June 2019. The study was approved by the institutional ethics committee (158200-16-843-357).

### 2.2. Inclusion and Exclusion Criteria

Women with an early viable pregnancy (less than 12 weeks’ gestation) between the ages of 18 and 45 years who gave written informed consent were included in this study. All other women who did not meet all inclusion criteria were excluded.

### 2.3. Study Visits and Data Collection

Each participant experienced a total of three visits during the study: during the first trimester of pregnancy (<12 weeks of gestation), the second trimester of pregnancy (18–20 weeks of gestation), and two months after childbirth.

The first visit coincided with enrolment in this study, during which a detailed questionnaire was completed based on a comprehensive review of previous reports. It included demographic factors, physical activity and dietary data, anthropometric maternal measurements, and obstetrical and coloproctological anamnesis.

During each visit, a proctology questionnaire was completed. It included the most common symptoms of anal disorders (pain, bleeding from the anus, lumps in the anus, constipation and its type, and fecal and gas incontinence) and the most common anal disorders—HD, anal fissures, and constipation. Additionally, a physical examination was performed. A gynecologist was prepared by a colorectal surgeon to recognize and diagnose the perianal pathology using a standardized methodology before the study began. Constipation was defined by the Rome III criteria. Birth and neonatal data were obtained from medical records at the third visit. The frequency of these risk factors in patients with HD (HD group) was compared with that in patients who did not have HD (control group). In addition, to exclude falsely undiagnosed perianal disease, we examined whether subjects had symptoms characteristic of these conditions even though they had not been diagnosed with HD or constipation. We combined the indicators “postpartum HD”, “symptoms characteristic of perianal disease”, and “constipation during pregnancy” and named the resulting indicator “perianal pathology”.

### 2.4. Sample Size Calculation

This was a non-probabilistic sample. A total of 405 pregnant women were screened for eligibility for this study. Of them, 194 were excluded: 182 declined to participate, 5 did not meet inclusion criteria (were <18 years of age), and 7 had spontaneous miscarriages. We presumed the baseline risk of HD during pregnancy to be 35% from our previous experience. Based on a statistical power of 80% and a level of significance set at 6%, we calculated the total sample size to be 210 mothers. The ratio of women who had HD and who did not was 73:137 = 0.53, so the groups of the study consisted of 73 and 137 participants, respectively. We also presumed the baseline risk of perianal pathology after childbirth to be 35%. Based on a statistical power of 80% and a level of significance set at 5%, we calculated the total sample size to be 208 mothers. The ratio of women who had perianal pathology and who did not was 120:91 = 1.32; the groups of this study consisted of 118 and 90 participants, respectively.

### 2.5. Statistical Analysis

We performed statistical data analysis with the software package R statistical V 4.2.2 (31 October 2022) (© The R Foundation for Statistical Computing), RStudio 2022.07.2 Build 576 © 2024–2022 RStudio, PBC, IBM SPSS Statistics V.23, G*Power V. 3.1.9.4 University of Duesseldorf, Germany).

In describing the subjects’ characteristics, quantitative variables were reported as the mean with standard deviation (SD), median, first quartile (Q1), and third quartile (Q3). Qualitative variables were reported in absolute numbers and percentages.

We used the Shapiro–Wilk test to test the normality assumption of the quantitative variables.

We used the nonparametric Mann–Whitney U test for quantitative data that did not meet the normality conditions for comparisons between two independent groups.

We used the Welch parametric F test and the Bayes factor as an additional measure of hypothesis validity for comparisons of three or more independent groups when the variables met the conditions of normality.

When comparing two groups of variables, we used the rank biserial correlation coefficient (rpb) to estimate the effect size between interval (discrete) quantitative variables that did not meet the conditions of normality. We considered the effect size to be small if rpb < 0.05, very small if 0.05 ≤ rpb < 0.20, small if 0.20 ≤ rpb < 0.30, medium if 0.30 ≤ rpb < 0.40, and large if rpb > 0.41.

When comparing two or more groups of nominal variables, we used Cramer’s V to estimate the effect size.

## 3. Results

A total of 405 pregnant women were screened for eligibility for this study. The analysis of risk factors related to HD and perianal pathology included 70 (33.2%) and 120 (56.9%) women, respectively, after excluding women with missing information regarding relevant variables. In total, 70 patients were diagnosed with postpartum HD, and only 2 (0.95%) of them had thrombosed HD.

The study groups’ baseline demographic, obstetric, and coloproctological parameters are shown in Table 1 and Table 2. There were a few minor differences between the groups. We did not find statistically significant differences between groups comparing postpartum HD in demographic and obstetric data. However, we discovered a statistically significant strong association between this condition and a history of HD (ES = 0.36 (CI 95% 24–100, *p* < 0.001). Women diagnosed with this disease more frequently felt perianal discomfort (ES = 0.20 (CI 95% 8–100, *p* = 0.002), pain (ES = 0.15 (CI 95% 0–100, *p* = 0.021), and lumps (ES = 0.30 (CI 95% 18–100, *p* < 0.001) during the study period.

The women in the group with perianal pathology had a higher BMI before pregnancy than the healthy women (ES = −0.26 (CI 95% −0.40, −0.11), *p* = 0.001). Additionally, most of them had a history of vaginal delivery (ES = 0.15 (CI 95% 0–100, *p* = 0.038) and perineal tears (ES = 0.15 (CI 95% 0–100, *p* = 0.019). They were more likely to have a history of HD (ES = 0.23 (CI 95% 11–100, *p* < 0.001), perianal discomfort (ES = 0.27 (CI 95% 15–100, *p* < 0.001), lumps (ES = 0.27 (CI 95% 15–100, *p* < 0.001), and constipation (ES = 0.20 (CI 95% 7–100, *p* = 0.003) during pregnancy and the period after childbirth. Otherwise, there were no other statistically significant differences between the groups.

The pregnancy outcomes of women who fully completed the study are shown in Table 3.

We found that vaginal delivery without support was more common among women in the group diagnosed with postpartum HD (62 (85.4%) vs. 92 (66.3%), *p* = 0.017). In addition, women in this group delivered larger newborns (54 cm vs. 53 cm, *p* = 0.018), and the median duration of their second labor, in minutes, was longer (25 min. vs. 36 min., *p* = 0.019). All dependencies were statistically significant and moderately strong.

Women diagnosed with perianal pathology delivered heavier (3655 g vs. 3476 g, *p* = 0.028) and larger (53 cm vs. 54 cm, *p* = 0.021) neonates, and their second stage of labor was longer (32 min. vs. 25 min., *p* = 0.04) than that of healthy women.

Women who had postpartum HD were statistically more likely to suffer from symptoms characteristic of perianal disease during their pregnancy (22 (16%) vs. 46 (63%), *p* < 0.001) and constipation (32 (27%) vs. 34 (50%), *p* < 0.001).

Statistically significant differences in dietary data were found between women diagnosed with postpartum HD and healthy pregnant women. They consumed eggs daily or more frequently (97 (75%) vs. 62 (87%)) compared to less frequently than several times per week (30 (23%) vs. 7 (9.9%), *p* = 0.046), respectively. They were more likely to consume fruits and vegetables several times per week (87 (65%) vs. 61 (85%)) than daily (46 (35%) vs. 11 (15%)), *p* = 0.003), respectively, and salted products several times per week (49 (38%) vs. 41 (56%)) compared to daily, respectively. A total of 41 (56%)) were more likely to consume products cooked in oil daily (54 (42%) vs. 20 (27%)) compared to never (27 (21%) vs. 12 (16%), *p* = 0.037) (88 (70%) vs. 53 (73%)) or several times per week (26 (21%) vs. 6 (8.2%), *p* = 0.02).

While constipation during pregnancy and postpartum HD are the most important factors influencing perianal pathology, we constructed a logistic regression prognostic model to predict this condition. Before that, we used Youden’s method to determine the critical values for the most important indicators that make up the optimized logistic equations (Table 4).

After constructing and optimizing the logistic equation for postpartum HD (Table 5), we obtained that a newborn weight greater than 3380 g increases the probability of this disease by 4.22 times compared to a newborn weight lower than 3380 g (OR 4.22, CI 95% 1.83–9.71, *p* < 0.001). Moreover, smoking increases the probability of this disease by 6,59 times compared to non-smoking (OR 6.59, CI 95% 0.95–46.01, *p* = 0.057). Some dietary habits were associated with a higher risk of postpartum HD: consumption of eggs several times per week increased the probability of disease by 3.1 times compared to daily consumption of those products (OR 3.10, CI 95% 1.31–8.53, *p* = 0.028) and consumption of grain several times per week increased this probability by 2,87 times compared to daily consumption (OR 2.87, CI 95% 1.32–6.25, *p* = 0.008). Consumption of fruits and vegetables several times per week had the opposite effect: it reduced the probability of development of this disease by 2,86 times compared to daily consumption (OR 0.35, CI 95% 0.15–0.81, *p* = 0.014).

After constructing and optimizing the logistic equation for perianal pathology (Table 6), we found that newborn weight greater than 3380 g increases the probability of this disease by 3.95 times compared to newborn weight lower than 3380 g (OR 3.95, CI 95% 1.47–10.59, *p* = 0.006). Moreover, a BMI of more than 21.48 increases the probability (OR 3.56, CI 95% 1.51–8.47, *p* = 0.004) of this disease. Some dietary habits were associated with a higher risk of postpartum HD: consumption of eggs several times per week decreased the probability of disease by 5.56 times compared to daily consumption of those products (OR 0.18, CI 95% 0.06–0.56, *p* = 0.003) and consumption of flour several times per week increased this probability by 2.77 times compared to daily consumption (OR 2.77, CI 95% 1.10–6.98, *p* = 0.030).

The logistic regression equation model for postpartum HD fitted the included indicators well (AU ROC 0.801, CI 95% 73–86%, *p* < 0.001, pseudo-R^2^ (Cragg–Uhler) = 0.33, pseudo-R^2^ (McFadden) = 0.21, sensitivity = 60%, CI 95% (48–72%), specificity = 84%, CI 95% (76–90%), positive prognostic value = 67%, CI 95% (54–78%), negative prognostic value = 79%, CI 95% (72–86%), and prevalence = 35%, CI 95% (29–42%).

The prognostic equation allows fairly accurate prediction of the development of anal pathology during pregnancy (AU ROC 0.821, χ^2^(10) = 48.05, *p* = 0.00, Pseudo-R^2^ (Cragg-Uhler) = 0.39, Pseudo-R^2^ (McFadden) = 0.26, sensitivity = 90%, CI 95% (81–95%), specificity = 59%, CI 95% (45–72%), positive prognostic value = 78%, CI 95% (68–86%), negative prognostic value = 78%, CI% (62–89%), prevalence = 61%, CI% (53–70%).

**Figure 1:**
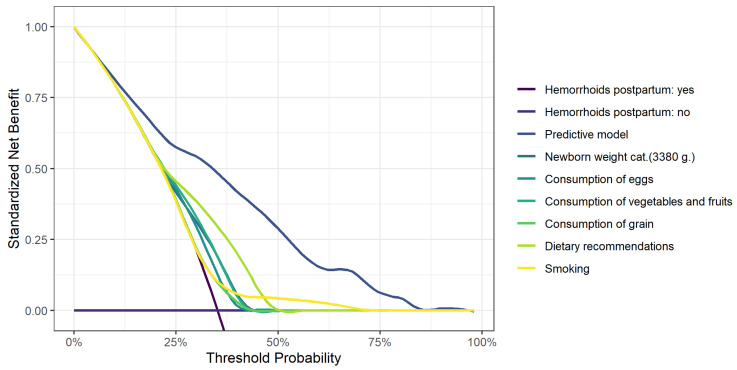
A decision curve analysis for postpartum hemorrhoidal disease.

**Figure 2:**
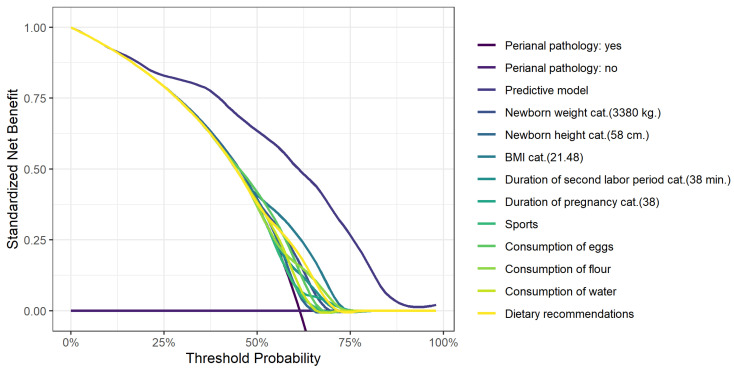
A decision curve analysis for the development of perianal pathology.

Figure 1 shows the decision curve analysis for postpartum HD and Figure 2 shows the decision curve analysis for perianal pathology.

## 4. Discussion

### 4.1. Main Findings

This study identified an incidence of perianal pathology of 61.4% and postpartum HD of 35.1%. Multivariate analysis identified a neonatal birth weight ≥3380 g (OR 4.22; CI 95% 1.83–9.71, *p* < 0.001) and consumption of eggs (OR 3.10; CI 95% 1.13–8.53, *p* = 0.028) or cereals (OR 2.87; CI 95% 1.32–6.25, *p* = 0.008) several times a week as significant risk factors for hemorrhoidal disease. Neonatal birth weight ≥3380 g (OR 3.95; CI 95% 1.47–10.59, *p* = 0.006), maternal BMI ≥ 21.48 (OR 3.58; CI 95% 1.51–8.47, *p* = 0.004), duration of the second labor period ≥ 38 min (OR 2.81; CI 95% 1.09–7.23, *p* = 0.032), and consumption of flour products several times per week (OR 2.77; CI 95% 1.10–6.98, *p* = 0.030) were associated with a higher risk of perianal pathology. Daily consumption of fruits and vegetables (OR 0.35; CI 95% 0.15–0.81, *p* = 0.014) and less frequent consumption of eggs were protective factors (OR 0.18; CI 95% 0.06–0-0.56, *p* = 0.003).

### 4.2. Strengths and Limitations

This study has significant strengths. For instance, this was a prospective cohort study that was conducted in three institutions. The main weak point of the study was that we used only questionnaire assessment and physical examination and no other perianal pathology diagnostic procedures like colonoscopy. However, our aim was to avoid any unnecessary interventions as our study population was pregnant and postpartum women. Moreover, all participants who developed perianal symptoms were consulted by a colorectal surgeon and anoscopy was performed. Another potential limitation may be the relatively small sample size.

### 4.3. Interpretation

Maternal perianal pathologies, especially HD, are common during pregnancy and after delivery. They affect maternal health and quality of life. Although there are several studies on HD and perianal disease in the general population, these data are sparse for women during pregnancy and the postpartum period.

The incidence of postpartum HD in this study was 35.1%. This is consistent with the 43.9% rate observed by Buzinskiene and Poskus and the 38.1% rate observed in a similar population by Hong et al. [2,5,19]. The incidence of thrombosed external HD in our study was lower than that estimated by Ferdinande et al. (0.95% vs. 14.6%) [15].

The incidence of perianal pathology was 61.4%. Unadkat conducted a prospective study involving 217 pregnant women. They found that 27% of the participants had at least one symptom of perianal disease [20].

In the general population, constipation, a low-fiber diet, a high body mass index, pregnancy, and a sedentary lifestyle are defined as major risk factors for HD development [21]. Previous studies reported that Caucasians are affected 1.5 times more often [4]. We could not compare this risk factor because, in our study, all participants were Caucasian. Moreover, increased prevalence rates of HD were associated with higher socioeconomic status [4,19]. We did not find statistically significant differences in demographic data between groups with and without postpartum HD. A variety of diagnoses are associated with HD: depression, allergy, type I or II diabetes, Crohn’s disease, hematological diseases or blood cancer, psoriasis, asthma, varicose veins of the legs, restless leg syndrome, personal HD anamnesis, hypertension, spinal cord injury, and various diseases of the rectum and anus [22,23]. Although we did not confirm HD’s association with other diseases, we found a statistically significant strong association between this condition and a personal history of HD. Previous studies report overweight and obesity as HD risk factors [15,24,25,26]. Overweight persons are 2.6 times more likely to be ill [26]. In our study, women diagnosed with HD and perianal pathology had a higher BMI before pregnancy than the healthy ones. However, only a small number of participants developed these outcomes, and critical values for developing those pathologies were in normal BMI ranges (20.57 in the perianal pathology group and 21.48 in the postpartum HD group). This makes BMI a very questionable risk factor.

This study is the first study in which dietary impact on perianal pathology and HD is evaluated. We found that consumption of eggs (OR 3.10; CI 95% 1.13–8.53, *p* = 0.028) or cereals (OR 2.87; CI 95% 1.32–6.25, *p* = 0.008) several times a week were significant risk factors for developing HD. Moreover, consumption of flour products several times per week (OR 2.77; CI 95% 1.10–6.98, *p* = 0.030) was associated with a higher risk of perianal pathology. Daily consumption of fruits and vegetables (OR 0.35; CI 95% 0.15–0.81, *p* = 0.014) and less frequent consumption of eggs were protective factors (OR 0.18; CI 95% 0.06–0-0.56, *p* = 0.003). Those dietary changes can be easily recommended to pregnant and postpartum women to reduce the risk of those pathologies without causing any complications or negative outcomes.

Previous studies have compared women diagnosed with HD and perianal pathology with healthy pregnant women. Poskus (2014) conducted a prospective observational cohort study that surveyed 280 women who had given birth in Lithuania. In a multivariate analysis, he identified a personal history of perianal disease (OR 11.93; CI 95% 2.18–65.30), constipation (OR 18.98; CI 95% 7.13–50.54), straining during labor for more than 20 min (OR 29.75; CI 95% 4.00–221.23), and newborn birth weight >3800 g (OR 17.99; CI 95% 3.29–98.49) as significant predictors of HD and anal fissures during pregnancy and the perinatal period [5]. We found that lower neonatal weight (3380 g) may also be a risk factor for developing HD after birth. However, only a small number of participants developed the outcome, which makes it a very questionable risk factor.

However, we did not observe that constipation before the first trimester and a history of perianal disease were associated with HD after delivery. Moreover, the duration of the second birth period in our study was longer and was not estimated for the development of HD but rather for perianal pathology.

Abramowitz (2002) compared 165 women during the last 3 months of pregnancy and after delivery (within 2 months). The independent risk factors for anal lesions were dyschezia, with an odds ratio of 5.7 (CI 95% 2.7–12), and late delivery, with an odds ratio of 1.4 (CI 95% 1.05–1.9). Furthermore, thrombosed external HD was often observed after superficial perineal tears and in heavier babies (*p* < 0.05). Only 1 of the 33 patients with thrombosed external HD underwent Cesarean section [6]. The results of our study show that vaginal delivery without assistance was more prevalent among women in the group diagnosed with postpartum HD. However, we found no association between this condition and perineal tears.

Ferdinande (2018) found that 68% of 94 patients developed anal symptoms. The most common diagnoses were hemorrhoidal thrombosis (immediately after birth), hemorrhoidal prolapse (in the third trimester and immediately after birth), and anal fissure (not episode-related). The two independent risk factors for anal symptoms were constipation, with an odds ratio of 6.3 (CI 95% 2.08–19.37), and a history of anal problems, with an odds ratio of 3.9 (CI 95% 1.2–13) [15]. The results of our study were similar: women who had postpartum HD were more likely to suffer symptoms characteristic of perianal disease and constipation during their pregnancy.

Our results confirm that bigger neonatal weight is a risk factor for the development of HD and perianal pathology. Our data also show that length of labor and maternal BMI before pregnancy are risk factors for the development of perianal pathology. Our final independent risk factor for postpartum HD, more frequent consumption of eggs and cereals, has not yet been demonstrated and requires additional investigation. Based on our findings, we recommend paying more attention to normal maternal body mass index and eating habits while planning and during pregnancy. Counseling about healthy eating and keeping physically active during pregnancy is essential for preventing various health disorders. The results of our study show that perianal pathology and especially HD are also associated with healthy living habits. However, future research should consider the potential effects of maternal BMI and neonatal weight more carefully since, in our study, only a small number of participants developed the outcome, which makes them very questionable risk factors. Moreover, future research could examine the association between eating habits and perianal pathology since those diseases affect almost half of all pregnant women and some dietary interventions could reverse this trend. This research indicates the necessity of monitoring and evaluating dietary behaviors, which will enable the early diagnosis and prevention of perianal diseases.

## 5. Conclusions

Perianal pathology and especially HD are common during pregnancy and the postpartum period. In conclusion, neonatal birth weight ≥ 3380 g and daily consumption of eggs and cereals were identified as independent risk factors for postpartum HD. A newborn birth weight of ≥3380 g, a maternal BMI of ≥21.48, duration of the second labor period of ≥38 min, and consumption of cereals several times per week increased the likelihood of developing perianal pathology. Daily consumption of fruits and vegetables and less frequent consumption of eggs could be protective factors for perianal pathology development. Future studies should aim to replicate these results in a larger sample.

## Figures and Tables

**Table 1:** Baseline characteristics by groups with and without postpartum hemorrhoidal disease.

	No PHD * (N = 138)	PHD * (N = 73)	*p*-Value **	ES *** (95% CI)
Demographic variables				
Age [mean ± SD]	30.3 ±4.4	30.9 ±4.4	0.2	−0.12 (−0.27, 0.05)
Median [Q1, Q3]	30.0 [27.0, 32.0]	31.0 [27.0, 34.0]		
BMI (before pregnancy) [mean ± SD]	21.5 [20.1, 24.2]	22.0 [20.9, 24.6]	0.12	−0.13 (−0.29, 0.03)
Median [Q1, Q3]	21.5 [20.1, 24.2]	22.0 [20.9, 24.6]		
BMI evaluation (before pregnancy) [n (%)]			0.5	0.00 (0.00, 1.0)
Too low	13 (9.4%)	3 (4.1%)		
Normal	97 (70.0%)	54 (74.0%)		
Overweight	20 (14.0%)	10 (14.0%)		
Obese	8 (5.8%)	6 (8.2%)		
Marital status [n (%)]			0.7	0.00 (0.00, 1.0)
Married	106 (77.0%)	61 (84.0%)		
Partnership	23 (17.0%)	9 (12.0%)		
Single	8 (5.8%)	3 (4.1%)		
Education [n (%)]			0.8	0.00 (0.00, 1.0)
Secondary	16 (12.0%)	6 (8.2%)		
Special secondary	19 (14.0%)	8 (11.0%)		
Unfinished higher	16 (12.0%)	8 (11.0%)		
Higher	87 (63.0%)	51 (70.0%)		
Living conditions [n (%)]			0.7	0.00 (0.00, 1.0)
Satisfactory	23 (17.0%)	14 (19.0%)		
Good	115 (83.0%)	59 (81.0%)		
Living area [n (%)]			0.6	0.00 (0.00, 1.0)
Rural	31 (22.0%)	14 (19.0%)		
Urban	107 (78.0%)	59 (81.0%)		
Monthly income [n (%)]			0.7	0.00 (0.00, 1.0)
<EUR 300	12 (8.7%)	4 (5.5%)		
EUR 300–500	31 (22.0%)	18 (25.0%)		
>EUR 500	95 (69.0%)	51 (70.0%)		
Physical activity [n (%)]			0.6	0.00 (0.00, 1.0)
Too low	77 (56.0%)	35 (48.0%)		
Enough	61 (44.4%)	36 (49.4%)		
Sports [n (%)]			0.076	0.10 (0.00, 1.0)
No	40 (29.0%)	30 (41.0%)		
Yes	98 (71.0%)	43 (59.0%)		
Obstetric variables				
Menarche [mean ± SD]	11.6 ± 4.5	12.2 ± 4.0	0.4	−0.07 (−0.23, 0.10)
Median [Q1, Q3]	13.0 [12.0, 14.0]	13.0 [12.0, 14.0]		
Number of previous pregnancies [n (%)]			0.4	0.00 (0.00, 1.0)
0	66 (48.0%)	29 (40.0%)		
1	41 (30.0%)	24 (33.0%)		
2	26 (19.0%)	14 (19.0%)		
3 and more	5 (3.6%)	6 (8.2%)		
Outcomes of previous delivery [n (%)]			0.4	0.00 (0.00, 1.0)
Did not give birth	75 (54.0%)	37 (51.0%)		
Vaginal delivery	47 (33.7%)	32 (42.8%)		
Cesarean delivery	16 (12.0%)	5 (6.8%)		
Previous vaginal tear [n (%)]			0.7	0.00 (0.00, 1.0)
No	132 (96.0%)	69 (95.0%)		
Yes	6 (4.3%)	4 (5.5%)		
Previous perineal tear [n (%)]			0.2	0.07 (0.00, 1.0)
No	115 (83.0%)	55 (75.0%)		
Yes	23 (17.0%)	18 (25.0%)		
Previous episiotomy [n (%)]			0.4	0.00 (0.00, 1.0)
No	94 (69.0%)	54 (74.0%)		
Yes	43 (31.0%)	19 (26.0%)		
Coloproctological variables				
History of HD [n (%)]			<0.001	0.36 (0.24, 1.0)
No	136 (99.0%)	56 (77.0%)		
Yes	2 (1.4%)	17 (23.0%)		
Current perianal discomfort [n (%)]			0.002	0.20 (0.08, 1.0)
No	118 (86.0%)	49 (67.0%)		
Yes	20 (14.0%)	24 (33.0%)		
Current perianal pain [n (%)]			0.021	0.15 (0.00, 1.0)
No	133 (96.0%)	64 (88.0%)		
Yes	5 (3.6%)	9 (12.0%)		
Current perianal bleeding [n (%)]			0.051	0.13 (0.00, 1.0)
No	134 (97.0%)	66 (90.0%)		
Yes	4 (2.9%)	7 (9.6%)		
Current perianal lumps [n (%)]			<0.001	0.30 (0.18, 1.0)
No	134 (97.0%)	57 (78.0%)		
Yes	4 (2.9%)	16 (22.0%)		
Constipation [n (%)]			0.11	0.08 (0.00, 1.0)
No	121 (88.0%)	58 (79.0%)		
Yes	17 (12.0%)	15 (21.0%)		
History of perianal operations [n (%)]			0.11	0.12 (0.00, 1.0)
No	138 (100%)	69 (97.0%)		
Yes	0 (0.0%)	2 (2.8%)		
Family history of perianal disease [n (%)]			0.4	0.00 (0.00, 1.0)
No	114 (83.0%)	57 (78.0%)		
Yes	24 (17.0%)	16 (22.0%)		

* PHD—postpartum hemorrhoidal disease. ** Wilcoxon rank sum test; Fisher’s exact test; Pearson’s Chi-squared test. *** Cramer’s V effect size; rank biserial correlation coefficient (ES—effect size).

**Table 2:** Baseline characteristics by groups with and without perianal pathology after childbirth.

	No PP * (N = 91)	PP * (N = 120)	*p*-Value **	ES *** (95% CI)
Demographic variables				
Age [mean ± SD]	30.3 ± 4.4	30.3 ± 4.5	>0.9	0.00 (−0.16, 0.15)
Median [Q1, Q3]	31.0 [27.5, 32.5]	30.0 [27.0, 34.0]		
BMI (before pregnancy) [mean ± SD]	22.6 ± 4.1	23.1 ± 3.4	0.001	−0.26 (−0.40, −0.11)
median [Q1, Q3]	21.4 [19.8, 24.4]	22.1 [20.9, 24.5]		
BMI evaluation (before pregnancy) [n (%)]			0.5	0.00 (0.00, 1.0)
Too low	14 (9.9%)	2 (2.9%)		
Normal	97 (69%)	52 (76%)		
Overweight	19 (13%)	11 (16%)		
Obese	11 (7.8%)	3 (4.4%)		
Marital status [n (%)]			0.2	0.08 (0.00, 1.0)
Married	69 (76.0%)	98 (82.0%)		
Partnership	18 (20.0%)	14 (12.0%)		
Single	4 (4.4%)	8 (6.6%)		
Education [n (%)]			>0.9	0.00 (0.00, 1.0)
Secondary	10 (11.0%)	12 (10.0%)	0.3	0.05 (0.00, 1.0)
Special secondary	10 (11.0%)	13 (11.0%)		
Unfinished higher	11 (12.0%)	13 (11.0%)		
Higher	60 (66.0%)	78 (65.0%)		
Living conditions [n (%)]			0.6	0.00 (0.00, 1.0)
Satisfactory	14 (15.0%)	23 (19.0%)		
Good	77 (85.0%)	97 (81.0%)		
Living area [n (%)]			0.6	0.00 (0.00, 1.0)
Rural	18 (20.0%)	27 (22.0%)		
Urban	73 (80.0%)	93 (78.0%)		
Monthly income [n (%)]			0.13	0.10 (0.00, 1.0)
<EUR 300	8 (8.8%)	8 (6.7%)		
EUR 300–500	15 (16.0%)	34 (28.0%)		
>EUR 500	68 (75.0%)	78 (65.0%)		
Physical activity [n (%)]			0.7	0.00 (0.00, 1.0)
Too low	48 (53.0%)	64 (53.0%)		
Enough	43 (47.2%)	56 (46.8%)		
Sports [n (%)]			0.13	0.00 (0.00, 1.0)
No	25 (27.0%)	45 (38.0%)		
Yes	66 (73.0%)	75 (62.0%)		
Obstetric variables				
Menarche [mean ± SD]	11.2 ± 4.8	12.2 ± 3.8	0.3	−0.09 (−0.24, 0.07)
median [Q1, Q3]	13.0 [11.5, 14.0]	13.0 [12.0, 14.0]		
Number of previous pregnancies [n (%)]			0.075	0.14 (0.00, 1.0)
0	50 (55.0%)	45 (38.0%)		
1	25 (27.0%)	40 (33.0%)		
2	13 (14.0%)	27 (22.0%)		
3 and more	11 (5.2%)	8 (6.7%)		
Outcomes of previous delivery [n (%)]			0.038	0.15 (0.00, 1.0)
Did not give birth	56 (62.0%)	56 (47.0%)		
Vaginal delivery	24 (26.0%)	54 (44.5%)		
Cesarean delivery	11 (12.0%)	10 (8.3%)		
Previous vaginal tear [n (%)]			0.7	0.00 (0.00, 1.0)
No	86 (95.0%)	115 (96.0%)		
Yes	5 (5.5%)	5 (4.2%)		
Previous perineal tear [n (%)]			0.019	0.15 (0.00, 1.0)
No	80 (88.0%)	90 (75.0%)		
Yes	11 (12.0%)	30 (25.0%)		
Previous episiotomy [n (%)]			0.7	0.00 (0.00, 1.0)
No	62 (69.0%)	86 (72.0%)		
Yes	28 (31.0%)	34 (28.0%)		
Coloproctological variables				
History of HD [n (%)]			<0.001	0.23 (0.11, 1.0)
No	90 (99.0%)	102 (85.0%)		
Yes	1 (1.1%)	18 (15.0%)		
Current perianal discomfort [n (%)]			<0.001	0.27 (0.15, 1.0)
No	84 (92.0%)	83 (69.0%)		
Yes	7 (7.7%)	37 (31.0%)		
Current perianal pain [n (%)]			0.09	0.09 (0.00, 1.0)
No	88 (97.0%)	109 (91.0%)		
Yes	3 (3.3%)	11 (9.2%)		
Current perianal bleeding [n (%)]			0.12	0.10 (0.00, 1.0)
No	89 (98.0%)	111 (92.0%)		
Yes	2 (2.2%)	9 (7.5%)		
Current perianal lumps [n (%)]			<0.001	0.27 (0.15, 1.0)
No	91 (100%)	100 (83.0%)		
Yes	0 (0.0%)	20 (17.0%)		
Constipation [n (%)]			0.003	0.20 (0.07, 1.0)
No	85 (93.0%)	94 (78.0%)		
Yes	6 (6.6%)	26 (22.0%)		
History of perianal operations [n (%)]			0.5	0.05 (0.00, 1.0)
No	91 (100%)	116 (98.0%)		
Yes	0 (0.0%)	2 (1.7%)		
Family history of perianal disease [n (%)]			0.7	0.00 (0.00, 1.0)
No	75 (82.0%)	96 (80.0%)		
Yes	16 (18.0%)	24 (20.0%)		

* PP—perianal pathology. ** Wilcoxon rank sum test; Fisher’s exact test; Pearson’s Chi-squared test. *** Cramer’s V effect size; rank biserial correlation coefficient (ES—effect size).

**Table 3:** Pregnancy characteristics by groups according to the presence of postpartum hemorrhoidal disease and perianal pathology.

HDP *				
	No HDP * (N = 138)	HDP * (N = 73)	*p*-Value **	ES *** (95% CI)
Birth week [mean ± SD]	38.78 ± 2.17	38.97 ± 1.78	0.8	−0.02 (−0.18, 0.14)
Median [Q1, Q3]	39.0 [39.0, 40.0]	39.0 [38.0, 40.0]		
Preterm birth [n (%)]			0.3	0.02 (0.00, 1.0)
Yes	18 (13.0%)	6 (8.2%)		
No	120 (87.0%)	67 (92.0%)		
Birth assistance [n (%)]			0.017	0.19 (0.00, 1.0)
Vaginal birth without assistance	92 (66.3%)	62 (85.4%)		
Vaginal birth with assistance	8 (5.8%)	2 (2.7%)		
Cesarean delivery	38 (28.0%)	9 (12.0%)		
Newborn weight [mean ± SD]	3375 ± 722	3586 ± 509	0.05	−0.16 (−0.32, 0.00)
Median [Q1, Q3]	3500.0 [3100.0, 3855.0]	3610.0 [3380.0, 3870.0]		
Newborn height [mean ± SD]	51.7 ± 4.5	53.2 ± 2.9	0.018	−0.20 (−0.35, −0.04)
Median [Q1, Q3]	53.0 [51.0, 55.0]	54.0 [52.0, 55.0]		
Head circumference [mean ± SD]	34.72 ± 2.13	35.27 ± 1.78	0.055	−0.16 (−0.31, 0.01)
Median [Q1, Q3]	35.0 [34.0, 36.0]	36.0 [34.0, 36.0]		
Duration of second labor period [mean ± SD]	31 ± 34	39 ± 30	0.019	−0.22 (−0.38, −0.04)
Median [Q1, Q3]	25.0 [2, 44]	36.0 [16, 52]		
PP ****				
	No PP **** (N = 91)	PP **** (N = 120)	*p*-value **	ES *** (95% CI)
Birth week [mean ± SD]	39.0 [38.0, 40.0]	39.5 [39.0, 40.0]	0.12	−0.13 (−0.29, 0.04)
Median [Q1, Q3]				
Preterm birth [n (%)]			0.2	0.06 (0.00, 1.0)
Yes	19 (13.0%)	5 (7.4%)		
No	122 (87.0%)	64 (93.0%)		
Birth assistance [n (%)]			0.1	0.12 (0.00, 1.0)
Vaginal birth without assistance	96 (68.1%)	57 (83.9%)		
Vaginal birth with assistance	8 (5.6%)	2 (3.0%)		
Cesarean delivery	37 (26.0%)	9 (13.0%)		
Newborn weight [mean ± SD]	3383 ± 694	3586 ± 579	0.028	−0.19 (−0.34, 0.02)
Median [Q1, Q3]	3476 [3130.0, 3840.0]	3655.0 [3322.0, 3975.0]		
Newborn height [mean ± SD]	51.8 ± 4.3	53.0 ± 3.4	0.021	−0.20 (−0.35, 0.03)
Median [Q1, Q3]	53.0 [51.0, 54.0]	54.0 [52.0, 55.0]		
Head circumference [mean ± SD]	34.86 ± 2.08	35.03 ± 1.95	0.6	−0.04 (−0.20, 0.13)
Median [Q1, Q3]	35.0 [34.0, 36.0]	35.0 [34.0, 36.0]		
Duration of second labor period [mean ± SD]	31 ± 32	40 ± 34	0.04	−0.20 (−0.37, −0.01)
Median [Q1, Q3]	25.0 [3, 44]	32.0 [19, 52]		

* PHD—postpartum hemorrhoidal disease. ** Wilcoxon rank sum test; Fisher’s exact test; Pearson’s Chi-squared test. *** Cramer’s V effect size; rank biserial correlation coefficient (ES—effect size). **** PP—perianal pathology.

**Table 4:** Critical points for the development of perianal pathology and postpartum hemorrhoidal disease.

Variable	Critical Value of the PHD *	Critical Value of the PP **
Newborn weight (g)	3380	3380
Newborn height (cm)	52	58
Duration of the second labor period (min)	38	38
Maternal BMI before pregnancy	20.57	21.48
Duration of pregnancy (wks)	41	38

* PHD—postpartum hemorrhoidal disease. ** PP—perianal pathology.

**Table 5:** Coefficients of the regression analysis of postpartum hemorrhoidal disease.

Variable	Value	HDP *		OR ** (Univariate Analysis)	OR ** (Multivariate Analysis)
		No, N = 129	Yes, N = 70		
Average newborn weight	<3380	54 (41.9%)	16 (22.9%)		
	≥3380	75 (58.1%)	54 (77.1%)	2.43 (1.26–4.69, *p* = 0.008)	4.22 (1.83–9.71, *p* < 0.001)
Consumption of eggs	daily or more frequently	30 (23.3%)	7 (10%)		
	several times a week	97 (75.2%)	61 (87.1%)	2.70 (1.11–6.52, *p* = 0.028)	3.10 (1.13–8.53, *p* = 0.028)
	never	2 (1.6%)	2 (2.9%)	4.29 (0.51–35.91, *p* = 0.180)	5.45 (0.46–64.31, *p* = 0.178)
Consumption of fruits and vegetables	daily or more frequently	84 (65.1%)	59 (84.3%)		
	several times a week	45 (34.9%)	11 (15.7%)	0.35 (0.17–0.73, *p* = 0.005)	0.35 (0.15–0.81, *p* = 0.014)
Consumption of grain	daily or more frequently	86 (66.7%)	40 (57.1%)		
	several times a week	42 (32.6%)	30 (42.9%)	1.54 (0.84–2.80, *p* = 0.161)	2.87 (1.32–6.25, *p* = 0.008)
	never	1 (0.8%)	0 (0%)	0.00 (0.00, *p* = 0.988)	0.00 (0.00, *p* = 0.990)
Smoking	no	95 (73.6%)	44 (62.9%)		
	smoked before	32 (24.8%)	21 (30%)	1.42 (0.74–2.73, *p* = 0.298)	1.85 (0.84–4.08, *p* = 0.126)
	yes	2 (1.6%)	5 (7.1%)	5.40 (1.01–28.91, *p* = 0.049)	6.59 (0.95–46.01, *p* = 0.057)

* PHD—postpartum hemorrhoidal disease. ** OR—odds ratio.

**Table 6:** Coefficients of the regression analysis of perianal pathology.

Variable	Value	PP *		OR ** (Univariate Analysis)	OR ** (Multivariate Analysis)
		No, N = 54	Yes, N = 86		
Average newborn weight	<3380	23 (42.6%)	22 (25.6%)		
	≥3380	31 (57.4%)	64 (74.4%)	2.16 (1.05–4.46, *p* = 0.037)	3.95 (1.47–10.59, *p* = 0.006)
Average newborn height	<58	51 (94.4%)	85 (98.8%)		
	≥58	3 (5.6%)	1 (1.2%)	0.20 (0.02–1.97, *p* = 0.168)	0.03 (0.00–0.47, *p* = 0.013)
BMI ***	<21.48	32 (59.3%)	29 (33.7%)		
	≥21.48	22 (40.7%)	57 (66.3%)	2.86 (1.42–5.78, *p* = 0.003)	3.58 (1.51–8.47, *p* = 0.004)
Duration of second labor period	<38	38 (70.4%)	49 (57%)		
	≥38	16 (29.6%)	37 (43%)	1.79 (0.87–3.70, *p* = 0.114)	2.81 (1.09–7.23, *p* = 0.032)
Gestation	<38	5 (9.3%)	14 (16.3%)		
	≥38	49 (90.7%)	72 (83.7%)	0.52 (0.18–1.55, *p* = 0.244)	0.09 (0.02–0.39, *p* = 0.001)
Sports	no	16 (29.6%)	31 (36%)		
	yes	38 (70.4%)	55 (64%)	0.75 (0.36–1.55, *p* = 0.434)	0.41 (0.15–1.13, *p* = 0.085)
Consumption of eggs	daily or more frequently	40 (74.1%)	76 (88.4%)		
	several times a week	14 (25.9%)	10 (11.6%)	0.38 (0.15–0.92, *p* = 0.033)	0.18 (0.06–0.56, *p* = 0.003)
Consumption of flour products	daily or more frequently	40 (74.1%)	50 (58.1%)		
	several times a week	14 (25.9%)	36 (41.9%)	2.06 (0.98–4.33, *p* = 0.058)	2.77 (1.10–6.98, *p* = 0.030)
Consumption of water	more, than 2 l/d	43 (79.6%)	76 (88.4%)		
	less, than 2 l/d	11 (20.4%)	10 (11.6%)	0.51 (0.20–1.31, *p* = 0.163)	0.33 (0.10–1.15, *p* = 0.083)

* PHD—postpartum hemorrhoidal disease. ** OR—odds ratio. *** BMI—body mass index.

## Data Availability

The datasets used and analyzed during this study are available from the corresponding author upon reasonable request. All data relevant to the study are included in the article or uploaded as supplemental online information. Unidentified data underlying the findings reported in this article will be released to third parties upon written request to the corresponding author describing the intent of the data use and the full affiliation of the requesting organization. A data access agreement must be signed to gain access to the data.
